# Low Level of Low-Density Lipoprotein Receptor-Related Protein 1 Predicts an Unfavorable Prognosis of Hepatocellular Carcinoma after Curative Resection

**DOI:** 10.1371/journal.pone.0032775

**Published:** 2012-03-12

**Authors:** Xiao-Yong Huang, Guo-Ming Shi, Ranjan Prasad Devbhandari, Ai-Wu Ke, Yuwei Wang, Xiao-Ying Wang, Zheng Wang, Ying-Hong Shi, Yong-Sheng Xiao, Zhen-Bin Ding, Zhi Dai, Yang Xu, Wei-Ping Jia, Zhao-You Tang, Jia Fan, Jian Zhou

**Affiliations:** 1 Liver Cancer Institute, Fudan University, Zhongshan Hospital, Shanghai, People's Republic of China; 2 Shanghai Key Laboratory of Organ Transplantation, Fudan University, Zhongshan Hospital, Shanghai, People's Republic of China; 3 Department of Endocrinology and Metabolism, Shanghai Jiao Tong University, Affiliated Sixth People's Hospital, Shanghai, People's Republic of China; 4 Cancer Center, Institutes of Biomedical Sciences, Fudan University, Shanghai, People's Republic of China; 5 Key Laboratory of Carcinogenesis and Cancer Invasion, Fudan University, Ministry of Education, Shanghai, People's Republic of China,; University of Hong Kong, Hong Kong

## Abstract

**Background:**

Low-density lipoprotein receptor-related protein 1 (LRP1) is a multifunctional receptor involved in receptor-mediated endocytosis and cell signaling. The aim of this study was to elucidate the expression and mechanism of LRP1 in hepatocellular carcinoma (HCC).

**Methods:**

LRP1 expression in 4 HCC cell lines and 40 HCC samples was detected. After interruption of LRP1 expression in a HCC cell line either with specific lentiviral-mediated shRNA LRP1 or in the presence of the LRP1-specific chaperone, receptor-associated protein (RAP), the role of LRP1 in the migration and invasion of HCC cells was assessed in vivo and in vitro, and the expression of matrix metalloproteinase (MMP) 9 in cells and the bioactivity of MMP9 in the supernatant were assayed. The expression and prognostic value of LRP1 were investigated in 327 HCC specimens.

**Results:**

Low LRP1 expression was associated with poor HCC prognosis, with low expression independently related to shortened overall survival and increased tumor recurrence rate. Expression of LRP1 in non-recurrent HCC samples was significantly higher than that in early recurrent samples. LRP1 expression in HCC cell lines was inversely correlated with their metastatic potential. After inhibition of LRP1, low-metastatic SMCC-7721 cells showed enhanced migration and invasion and increased expression and bioactivity of MMP9. Correlation analysis showed a negative correlation between LRP1 and MMP9 expression in HCC patients. The prognostic value of LRP1 expression was validated in the independent data set.

**Conclusions:**

LRP1 modulated the level of MMP9 and low level of LRP1 expression was associated with aggressiveness and invasiveness in HCCs. LRP1 offered a possible strategy for tumor molecular therapy.

## Introduction

Hepatocellular carcinoma (HCC) is one of most frequent neoplasm worldwide [1], and has become a major cause of cancer-related death globally, owing to its high potential of invasion and metastasis. The molecular mechanism linked to invasion and metastasis of HCC is not fully understood. Hence, investigation of the underlying molecular mechanism may ultimately help in the development of innovative therapeutic strategies against HCC.

The low-density lipoprotein receptor (LDLR)-related protein-1 (LRP1) is a member of LDLR family, which is ubiquitously expressed in a variety of organs including adipose tissue, liver and brain [2]. It consists of a 515 kDa heavy chain that contains four clusters of ligand binding domains and a non-covalently associated 85 kDa light chain that contains a trans-membrane and cytoplasmic domain [3]. The biological activity of LRP1 was initially characterized as a clearance receptor for chylomicron remnants and complexes of α_2_-macroglobulin with proteinases [4]. Subsequent work has revealed that this receptor recognizes several classes of ligands, including serine proteinases, proteinase-inhibitor complexes, and the matricellular proteins TSP1 and TSP2 [5], [6], [7]. Recent studies indicate that LRP1 can bind a large number of cytoplasmic adaptor proteins via determinants located on its cytoplasmic domain in a phosphorylation-specific manner, and modulate the activity of other transmembrane receptors such as integrins and receptor tyrosine kinases [8]. Since the expression and activation of serine proteinases, urokinase plasminogen activator (uPA), TSP-1, TSP-2 as well as the matrix metalloproteinases (MMPs) can regulate the tumor microenvironment, the function of LRP1 as an endocytic receptor for diverse extracellular mediators may represent one mechanism by which LRP1 may regulate the tumor microenvironment and involve in tumor progression and spreading.

Although a growing number of studies have demonstrated that LRP1 is implicated in cancer progression, its precise role and potential underlying mechanism in specific cancers remain contentious [5]. Several studies have reported that low expression of LRP1 is closely related to aggressive tumor cells and advanced tumor stages, such as human endometrial carcinoma [9], thyroid cancer [10], Wilms tumors [11], lung cancer [12], breast and prostate cancer [13]. While, other studies argued that high LRP1 expression promotes breast cancer cell invasiveness, and LRP1 neutralization could abrogate cell motility in both tumor and nontumor cells despite the increased pericellular proteolytic activities of MMP2 and uPA [14]. Therefore, the LRP1 function in tumor cell migration and invasion likely depends on the tumor cell type and the specific extracellular proteins involved in these processes.

Recently, quantitative proteomics analysis of metastasis-related proteins in HCC cells showed a decrease of LRP1 level in MHCC-97H cell line with high metastasis potential, compared to low metastatic cell line MHCC-97L [15]. We used a combination of immunoprecipitation with mass spectrometry to develop an extensive protein–protein “interactome” network centered on tetraspanin CD151 in HCCLM3 cells, and identified LRP1 as an important molecular partner for CD151 with regard to metastasis of HCC [16], [17], [18], Therefore, LRP1 may play a specific role in the migration and invasion of HCC cells, probably relying on the specific molecular partner, which begs us for a closer look into the role of LRP1 in HCC. The present study demonstrates that low expression of LRP1 is a major contributor to the invasion-prone phenotype of HCC, and inhibition of LRP1, coupled to the increased expression and bioactivity of MMP9, enhances tumor cell migration and invasion. Our results also show that low level of LRP1 predicts an unfavorable prognosis of HCC after curative resection in the 2 independent patient cohorts.

## Materials and Methods

### Cell Lines and Animals

HCC cell lines Hep3B (low-metastatic human HCC cell line, American Type Culture Collection), SMMC-7721 (low-metastatic human HCC cell line, Chinese Academy of Science Cell Bank [19]), HCCLM3 and MHCC97L (human HCC cell lines with stepwise metastatic potential [20] established at the Liver Cancer Institute, Zhongshan Hospital, Fudan University) were used in this study. Male, athymic BALB/c nude mice (8 weeks old; Shanghai Institute of Material Medicine, Chinese Academy of Science, Shanghai, China) were raised under specific pathogen-free conditions. All animal work was performed in accordance with protocols approved by the Shanghai Medical Experimental Animal Care Commission. Ethical approval was obtained from the Research Ethics Committee of Zhongshan Hospital.

### Patients and Follow-up

Fresh HCC samples and their adjacent non-tumor samples were obtained from 327 consecutive patients who underwent curative HCC resection between 1997 and 2000 at the Liver Cancer Institute of Fudan University [21]. HCC diagnosis was based on World Health Organization criteria. Tumor differentiation was defined according to the Edmondson grading system [22]. Liver function was assessed using the Child–Pugh scoring system. Tumor staging was determined according to the sixth edition of the tumor–node–metastasis (TNM) classification of the International Union Against Cancer. Ethical approval was obtained from the Research Ethics Committee of Zhongshan Hospital, and written informed consent was obtained from each patient. Follow-up was terminated in March 2007. The median follow-up was 62 months (range, 4–121 months). The follow-up procedures were described in detail in our earlier study [21]. Treatment modalities after relapse were given according to a uniform guideline as described [21].

### RNA Extraction and Quantitative Real-time Polymerase Chain Reaction (qRT-PCR)

Four HCC cell lines and 40 HCC samples selected blindly from the above cohort, including 20 cases of HCC with early recurrence (within 2 years after curative resection) and 20 cases of HCC without early recurrence, were analyzed by qRT-PCR as described previously [17], with slight modification. Primers of β-actin as a control: sense: 5′-AGCGAGCATCCCCCAAAGTT-3′, anti-sense: 5′-GGGCACGAAGGCTCATCATT-3′. Primers of LRP1: 5′-ACATATAGCCTCCATCCTAATC-3′ and 5′-TTCCAATCTCCACGTTCAT-3′. Each sample was tested in triplicate. The mean Ct value for the β-actin gene was subtracted from the mean Ct value for LRP1 for each sample, using the following formula: LRP1ΔCt = (mean LRP1Ct−mean β-actin Ct). The fold change (2^−LRP1ΔCt^) of the LRP1 expression level relative to the β-actin expression level was calculated for each HCC sample.

### Immunoblotting and Immunofluorescence Assay

Thirty micrograms of total cell extract protein was run on sodium dodecyl sulfate-polyacrylamide gel electropheresis (SDS-PAGE), transferred onto polyvinylidene difluoride membranes, and incubated with the corresponding antibodies. The membranes were developed with the enhanced chemiluminescence method (Pierce, Rockford, IL, USA). Mouse anti-human LRP1 polyclonal antibody (1∶2000; Abnovus Biologicals UK) and rabbit anti-human MMP9 polyclonal antibody (1∶1000; Cell Signaling Technology, Danvers, MA, USA) was used to detect the expression of LRP1 and MMP9, respectively. GAPDH (1∶5,000; Chemicon, Temecula, CA, USA) was used as an internal control. All experiments were performed in triplicate. HCCLM3 and SMMC-7721 cells were used to detect the location of LRP1 by immunofluorescence assay as described previously [23]. The slices were assayed by fluorescence microscopy (Leica Microsystems Imaging Solutions, Cambridge, UK).

### Inhibition of LRP1: shRNA for LRP1 or receptor-associated protein(RAP)

Lentiviral-mediated pGCSIL-GFP-shRNA-LRP1 was constructed (Shanghai Genechem, Shanghai, China). We constructed 4 shRNA-LRP1 vectors (pGCSIL-GFP-shRNA-LRP1) to silence the expression of LRP1 in SMMC-7721 cells (SMMC-7721-vshLRP1). The most effective shRNA targeting sequence for LRP1 was as follows: 5′- CGGAGTGGTATTCTGGTATAA-3′. Stable transfectant clones were identified by qRT-PCR and immunoblotting. LRP1-specific chaperone, recombinant RAP(1 µM, novus Biologicals, USA) was preincubated with cells for 60 minutes at 37°C before the MMP9 expression and function analysis was performed.

### Cell Migration, Matrigel Invasion Assays and *In Vivo* Metastasis Assays

Cell migration and Matrigel invasion assays were performed as previously described [24]. A wound healing assay was used to evaluate the ability of cell migration. Cells grew to 80%–90% confluence in 24-well plates. A wound was made by dragging a plastic pipette tip across the cell surface. The remaining cells were washed three times to remove cell debris and incubated at 37°C with serum-free medium. At the indicated times, migrating cells at the wound front were photographed and compared. All experiments were performed in triplicate. Cell invasion assays were performed using 24-well transwells (8 µm pore size; Minipore) precoated with Matrigel (Falcon354480; BD Biosciences). Cells on the lower surface of the membrane were fixed in 4% paraformaldehyde and stained with Giemsa. Cells in 5 microscopic fields (magnification, ×200) were counted and photographed. All experiments were performed in triplicates.

The in vivo metastasis assays were performed as the previously described methods [16]. SMMC-7721-Mock and SMMC-7721-vshLRP1 cells (8.0×10^6^) were injected intrahepatically by a 27-gauge needle. Tumor volume was calculated using the following formula: V = π/6×length×width×height, and intrahepatic tumor and lung metastases of SMMC-7721-Mock and SMCC-7721-vshLRP1 were visualized with fluorescence stereomicroscopy (Leica Microsystems Imaging Solutions).

### Gelatin Zymography

The type IV collagenase activity of MMP9 in a conditioned medium was determined by gelatin zymography. Culture medium was prepared from either SMCC-7721, SMMC-7721-MOCK, SMMC-7721-vshLRP1 or SMMC-7721 treated with RAP cells. A total of 10^5^ cells were cultured in 1 ml of serum-free DMEM for 48 h, then culture media was electrophoresed at 4°C in 10% crosslinked SDS-PAGE, containing either 0.1% gelatin (Difco, Detroit, MI, USA). Following electrophoresis, the gel was washed with 2.5% Triton X-100 followed by incubation in Tris–HCl, 0.5 mM CaCl2, 10-6 M ZnCl2, pH 8.0, at 37°C for 16 h. Coomassie brilliant blue staining was then carried out.

### Construction of Tissue Microarrays and Immunohistochemistry

Tissue microarrays were constructed as described in our earlier study [21]. Immunohistochemical staining was performed as described elsewhere [24]. The intensity of LRP1- and MMP9-positive staining were measured mostly as described [21], based on a computerized image system, including a Leica DFC420 charge-coupled device camera and a Leica DM IRE2 microscope (Leica Microsystems Imaging Solutions). Briefly, three representative fields of each case were captured by the Leica QWin Plus v3 software under identical settings and magnification (×200). The area of positive staining in a photograph was measured by Image-Pro Plus v6.0software (Media Cybernetics, Inc.). The average proportion (area of positive staining/total area) on each spot (three images) was used to represent a particular sample. The expression of LRP1 and MMP9 was classified into two subgroups based on intensity, respectively (mean the average proportion as cutoff value, LRP1^high^, ≥45% of tumor section, and MMP9^high^, ≥20% of tumor section and LRP1^low^, <45%, and MMP9^low^, <20%).

### Statistical Analysis

Statistical analysis was performed with SPSS 12.0 software (SPSS, Chicago, IL, USA). Values are expressed as means ± standard deviation. Student's t test was used for comparison between groups. Correlation analysis was performed between LRP1 and MMP9. Overall survival (OS) and time to recurrence were defined as described previously [25]. OS and the cumulative recurrence rates were calculated by the Kaplan–Meier method and the log rank test. Cox's proportional hazards regression model was used to analyze the independent prognostic factors. P<0.05 was considered statistically significant.

### Independent validation

To further evaluate the prognostic performance of LRP1 expression, we validate in another independent cohort containing an additional series of 161 patients who underwent curative HCC resection in 2003 at the Liver Cancer Institute of Fudan University. Clinicopathologic features of this cohort of patients was described (Table S1). Immunohistochemistry, quantification of LRP1 expression, and statistics were conducted using the same methods.

## Results

### Low Expression of LRP1 was Correlated with High Metastatic Potential in HCC

**Figure 1:**
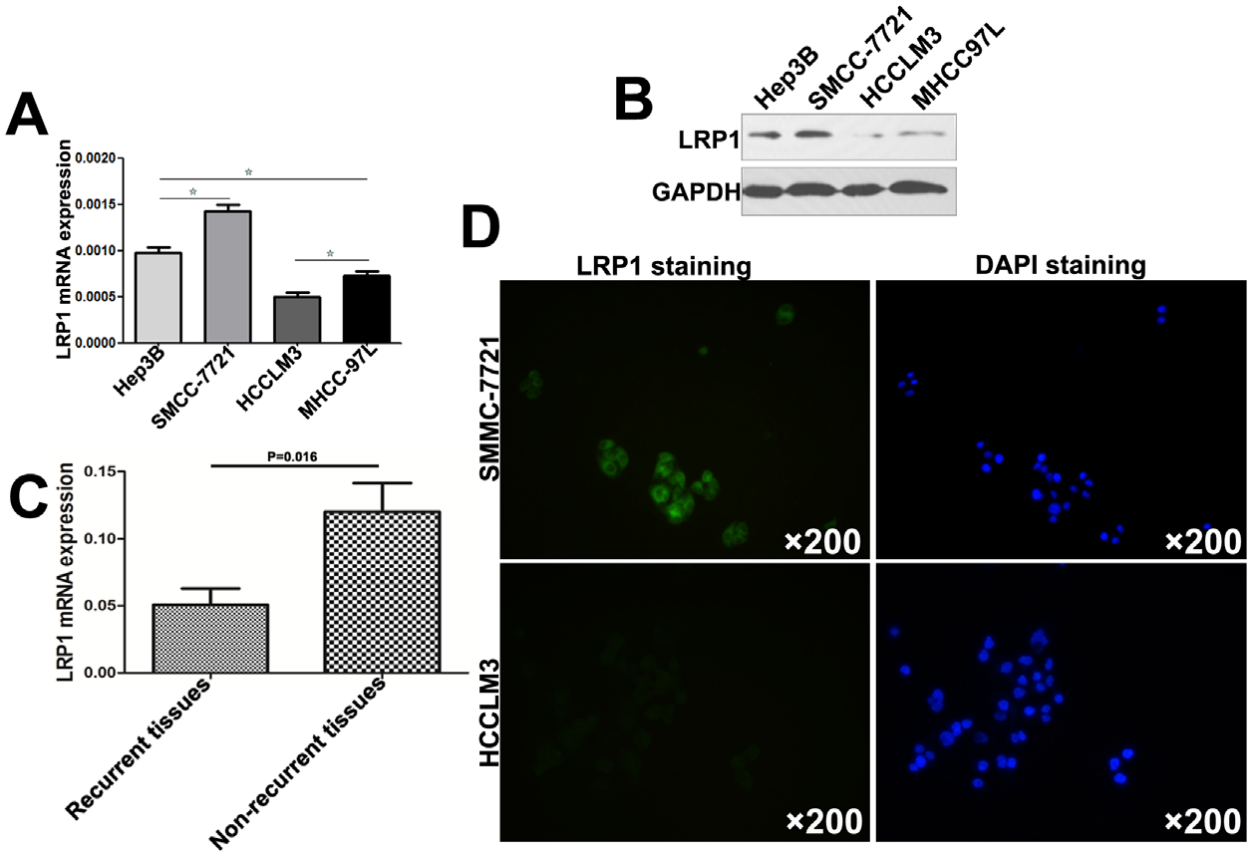
Expression and location of LRP1 in HCC cell lines and HCC tissues. Relative LRP1 mRNA levels (**A**) and protein levels (**B**) in Hep3B, SMMC-7721, HCCLM3 and MHCC-97L cells. (**C**) qRT-PCR showed LRP1 mRNA levels in HCC tissues with early recurrence were lower than that of HCC tissues without recurrence. (**D**) Fluorescence staining analysis for LRP1 expression in HCCLM3 and S MMC7721 cells.

LRP1 expression was detected in 4 HCC cell lines with different metastatic potential at the mRNA (Fig. 1A) and protein (Fig. 1B) levels. qRT-PCR showed that LRP1 expression level in the highest metastatic cell HCCLM3 was the lowest among 4 HCC cell lines (Fig. 1A, P<0.05), in line with the results from immunoblotting (Fig. 1B). We also examined the LRP1 mRNA expression in 40 cases of HCC. Strikingly, the LRP1 mRNA expression in non-recurrent HCC tissues (without recurrence within 2 years after curative resection) was 0.12±0.0047, which was higher than that in the early recurrence group (recurrence within 2 years after resection, 0.051±0.0027, P = 0.016, Fig. 1C). Immunofluorescence assay demonstrated that LRP1 localized on the cytoplasm membrane of HCCLM3 and SMMC-7721 cells (Fig. 1D). Immunofluorescence intensity of LRP1 in SMMC-7721 cells was stronger than that of HCCLM3 cells (Fig. 1D). The above data demonstrated that low LRP1 expression was related to the high metastatic potential in HCC.

### LRP1 Inhibition Up-regulated MMP9 Expression and Enhanced Mobility and Invasion of HCC Cell*s in vitro* and *in vivo*

**Figure 2:**
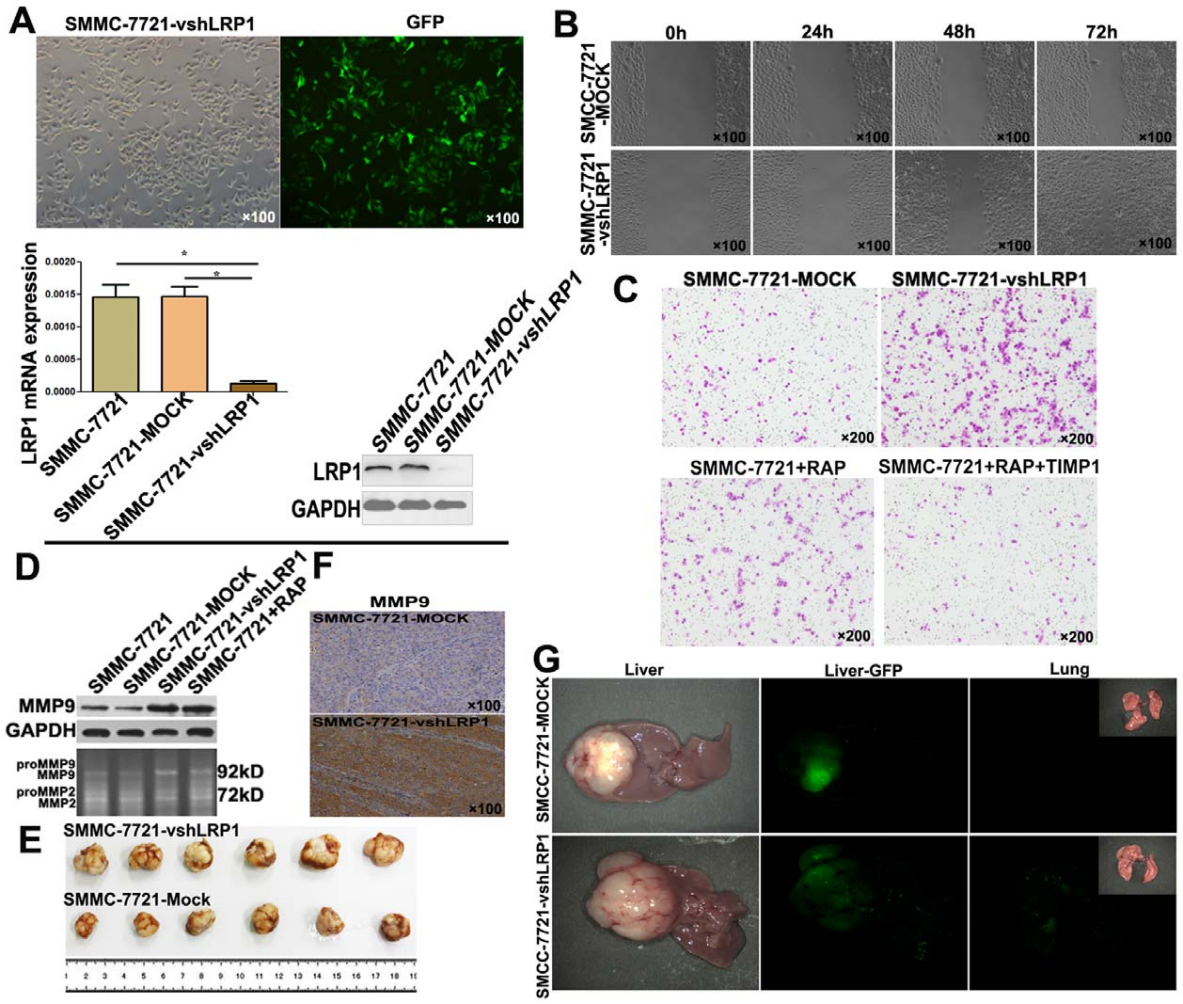
LRP1 inhibition enhanced invasion of SMCC-7721 cells and increased MMP9 expression and bioactivity *in vitro* and in *vivo*. (**A**) SMCC-7721 cells were successfully transfected with lentiviral-mediated pGCSIL-GFP-vshRNA-LRP1, and inhibition of LRP1 was validated by the qRT-PCR and immunoblottting. (**B**) wound healing assay, magnification ×100. (**C**) Transwell assay, magnification ×200. (**D**) The expression of MMP9 in cells and the bioactivity of MMP9 in the supernatant were assayed by western blot and gelatin zymography (lower panel), respectively. (**E**) The volume of SMMC-7721- vshLRP1-derived xenografts was larger than that of SMMC-7721-Mock-derived group. (F) Immunohistochemical staining for xenografts showed that down-regulation of LRP1 enhanced the level of MMP9 expression *in vivo*. (G) In the SMCC-7721-Mock xenografts, intrahepatic metastasis and lung metastasis were also markedly lower than those in the SMCC-7721-vshLRP1 groups.

We then determined the effect of LRP1 silencing on HCC cell mobility and invasion. LRP1 knockdown in SMCC-7721 cells was achieved by transfecting cells with pGCSIL-GFP-shRNA-LRP1 (Fig. 2A). Decreased expression of LRP1 in SMMC-7721 (>90%) was validated by qRT-PCR and Immunoblotting (Fig. 2A). Wound healing assay demonstrated accelerated wound closure in SMMC-7721-vshLRP1 cells, compared with SMMC-7721-Mock cells (Fig. 2B). Matrigel invasion assays showed markedly increased numbers of invaded SMMC-7721 cells after down-regulation of LRP1 expression using special shRNA (114.6±18.6 vs. 277.7±26.0, P = 0.001) (Fig. 2C). Increased number of invaded cells was also detected in SMMC-7721 cells blocked by LRP1-specific chaperone RAP (114.6±18.6 vs. 177.6±22.5, P = 0.019) (Fig. 2C). But when recombinant tissue inhibitor of metalloproteinases-1(TIMP-1, abcam, 25 µmol/L)) was applied to block MMP9 activity in SMMC-7721 cells treated with RAP, the number of invasive cells was significantly higher in the SMMC-7721 cells treated with RAP than the SMMC-7721 cells treated with TIMP-1 and RAP, suggesting that TIMP-1 could reverse the effect of LRP1 blockade by RAP (Fig. 2C). Immunoblotting and gelatin zymography revealed that MMP9 expression and bioactivity in SMMC-7721-vshLRP1 cells were enhanced when LRP1 in SMMC-7721 cells was down-regulated or blocked by RAP (Fig. 2D).

We then performed the *in vivo* metastasis assay to determine the metastasis potential of SMMC-7721 after LRP1 silencing. After successful formation of liver orthotropic tumors, tumor size and metastasis were assayed. The volume of SMMC-7721-Mock-derived and SMMC-7721-vshLRP1-derived xenografts were 0.827±0.440 and 1.758±0.503 cm^3^, respectively (P = 0.007, Fig. 2E). Immunohistochemical staining for xenografts showed that down-regulation of LRP1 also enhanced the level of MMP9 expression *in vivo* (Fig. 2F). The pulmonary metastasis rate in the SMMC-7721-vshLRP1 group was 83.3% (5/6), which was higher than that in the SMMC-7721-MOCK xenografts (16.6%, 1/6). In the SMCC-7721-Mock xenografts, intrahepatic metastasis were also markedly lower than those in the SMCC-7721-vshLRP1 groups (16.6% *vs.* 83.3%) (Fig. 2G).

### LRP1 Expression was a Beneficent Parameter for Predicting Prognosis in HCC Patients

**Figure 3:**
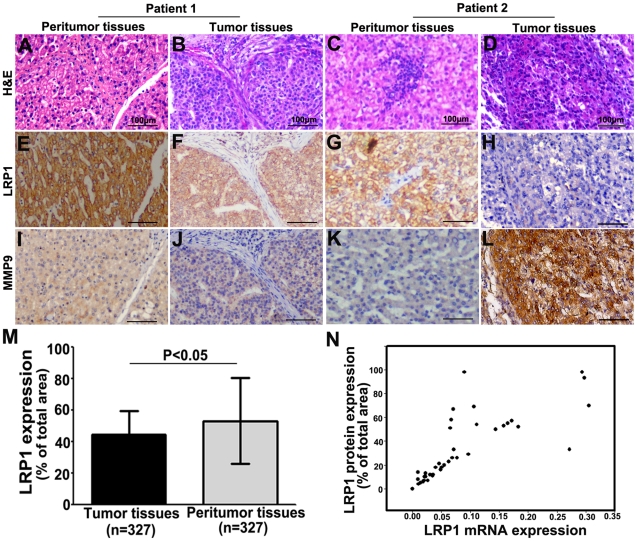
Expression of LRP1 and MMP9 in 327 cases of HCC. Hematoxylin & eosin staining of the tumor and corresponding peritumoral liver tissues (**A**, **B**, **C** and **D**). The LRP1 staining was mostly detected in the cell membrane of tumor cells, stromal cells and peritumoral liver cells(**E**, **F**, **G** and **H**). The expression of LRP1 protein had great variation in different tumor samples (**F** and **H**). the MMP9 protein was located in cytoplasm of tumor cells, peritumoral liver cells, stromal fibroblasts and inflammatory cells (**I**, **J**, **K** and **L**). Representative cases were listed. Patient 1 had high LRP1 expression and low expression of MMP9 (F and J), and patient 2 showed low LRP1 expression and high MMP9 expression in tumor tissue (H and L). The graph showed that the level of LRP1 protein expression was significantly down-regulated in tumors compared to that in the corresponding peritumoral liver tissues (**M**). A scatter plot showed that LRP1 protein expression in 40 tumor tissues blindly chosen from 327 cases of HCC was consistent with that of LRP1 mRNA (N). Scale bars: 100 µm.

After identification of primary HCC and peritumoral tissues using hematoxylin & eosin staining(Fig. 3A, B, C and D), expression of LRP1 protein was investigated in tissue microarrays consisting of 327 cases of HCC samples using immunohistochemistry (Fig. 3E, F, G and H). Immunoreactivity of LRP1 protein was observed in in the cell membranes of tumor cells, stromal cells and peritumoral liver cells (Fig. 3E, F, G and H). The expression of LRP protein in HCC cells had great variation in different tumor samples (Fig. 3. **F** and **H**). LRP1 protein expression in tumors was significantly lower than that in the corresponding peritumoral liver tissues in 327 cases of HCC (Fig. 3M; 44.8%±18.5% *vs.* 53.3%±27.1%, respectively, P<0.05, Fig. 3M). We have detected the expression of LRP1 mRNA in the above 40 HCC samples, and we compared the difference between LRP1 protein from immunohistochemistical staining and LRP1 mRNA in the same patients. A scatter plot revealed a significantly positive correlation between LRP1 protein and mRNA in 40 cases of HCC tissues (*r* = 0.769, P<0.001, Fig. 3N).

Of the 327 tumors, 161 (49.2%) were ranked as low and 166 (50.8%) as high LRP1 expression. LRP1^low^ was significantly correlated with vascular invasion (P = 0.037), none encapsulation (P = 0.002), high TNM staging (P = 0.043), and large tumor (P<0.001). However, other clinical characteristics, including age, sex, preoperative serum α-fetoprotein (AFP), liver cirrhosis, Child–Pugh score, preoperative treatment, tumor number and differentiation were not significantly related to the expression of LRP1 (Table 1).

**Table 1:** Correlation between LRP1 and clinicopathological characteristics in 327 HCCs.

Variables	LRP1 expression	*P* value
	% of Total area	
Sex		
Male	45.0±14.6	0.489
Female	43.5±14.2	
Age, years		
<52	44.3±15.2	0.534
≥52	45.3±14.0	
HBsAg		
Positive	44.9±14.3	0.776
Negative	44.3±15.7	
Liver cirrhosis		
Yes	44.7±14.5	0.839
No	45.2±15.2	
Preoperative treatment		
Yes	45.1±14.2	0.772
No	44.6±14.9	
Child–Pugh score		
A	44.8±14.5	0.824
B	42.5±27.6	
Serum AFP, ng/ml		
≤20	45.3±14.7	0.533
>20	44.3±14.5	
Tumor number		
Single	45.0±14.1	0.472
Multiple	43.4±16.9	
Microvascular invasion		
Yes	39.1±16.0	0.037
None	45.3±14.3	
Tumor encapsulation		
Complete	46.7±14.1	0.002
None	41.6±14.8	
Tumor differentiation		
I/II	45.8±14.4	0.069
III/IV	42.6±14.7	
Tumor diameter(cm)		
≤5	47.4±14.1	<0.001
>5	41.6±14.5	
TNM stage		
I/II	45.7±13.8	0.043
III	41.7±16.8	

Note: Values are expressed as the mean ± standard deviation.The student t test was used for comparison between groups. Abbreviations: AFP, α-fetoprotein; HBsAg, hepatitis B surface antigen. TNM, tumor-node-metastasis.

**Figure 4:**
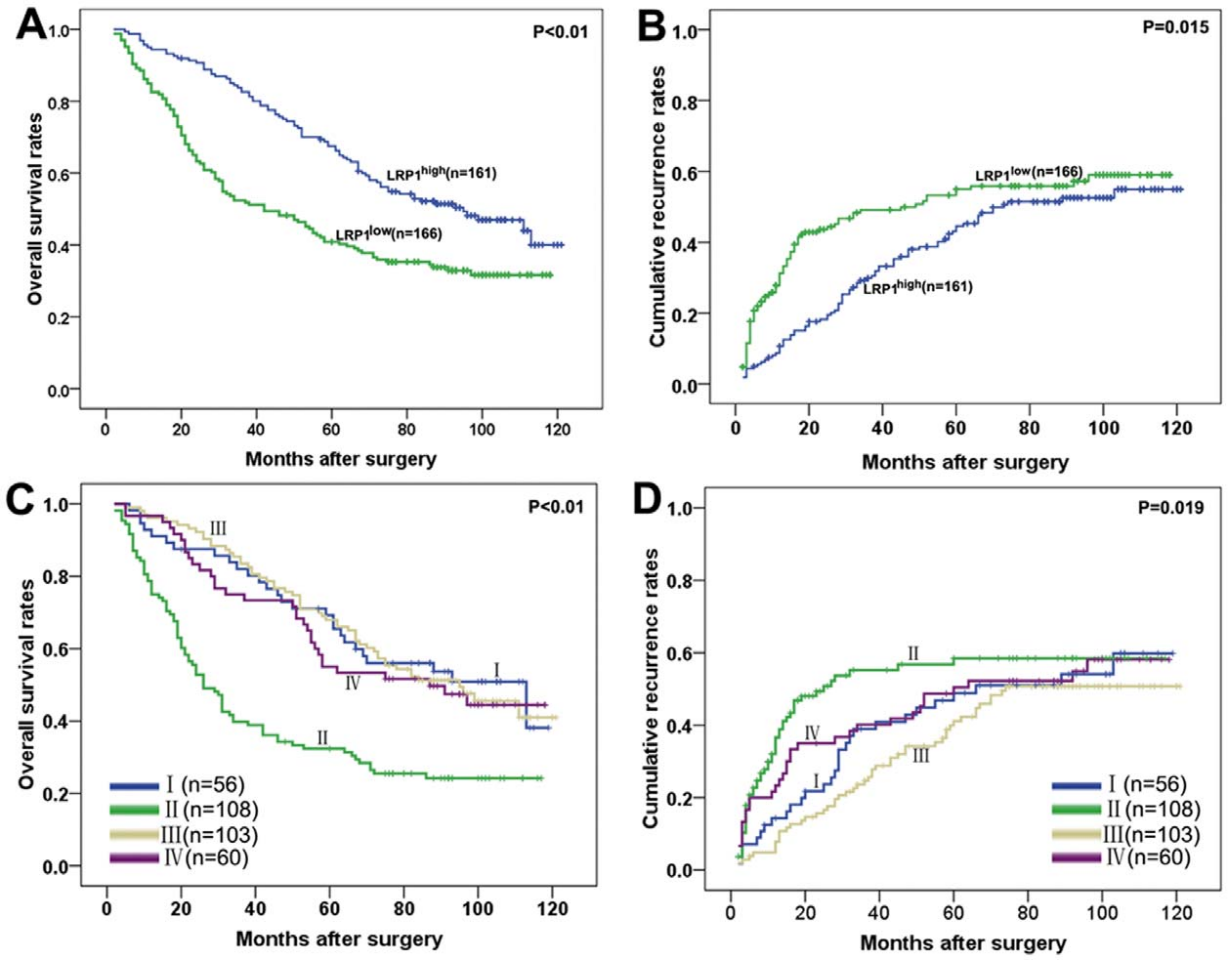
Prognostic significance was assessed by Kaplan–Meier analysis and log-rank tests. HCC patients with low LRP1 expression had poorer prognosis in terms of overall survival (**A**) and cumulative recurrence (**B**). HCC patients with LRP1^low^/MMP9^high^ showed the worst prognosis among the four subgroups (**C** and **D**, group I LRP1^high^/MMP9^high^ (n = 56), group II LRP1^low^/MMP9^high^ (n = 108), group III LRP1^high^/MMP9^low^ (n = 103), group IV LRP1^low^/MMP9^low^ (n = 60)).

The 3-, 5-, and 7-year OS rates in the whole cohort were 67.3%, 54.1% and 44.3% while cumulative recurrence were 36.7%, 45.6%, and 48.6%, respectively. Univariate analysis revealed that tumor size, tumor number, microvascular invasion, TNM staging and LRP1 expression were predictors for OS and cumulative recurrence. Tumor differentiation, tumor encapsulation and AFP were associated only with OS (Table 2). Individual clinicopathological features that showed significance by univariate analysis were adopted as covariates in a multivariate Cox proportional hazards model. LRP1 was an independent prognostic indicator for OS (P = 0.010) and cumulative recurrence (P = 0.031, Table 2). The 3-, 5-, and 7-year OS in the LRP1^low^ group was significantly lower than those in the LRP1^high^ group (52.4% *vs.* 83.8%, 40.9% *vs.* 67.5%, 35.3% *vs.* 52.2%, respectively, Fig. 4A). The 3-, 5-, and 7-year cumulative recurrence rates in the LRP1^high^ group were significantly lower than those in the LRP1^low^ group (29.9% *vs.* 49.1%, 44.5% *vs.* 51.4%, 47.8% *vs.* 55.0%, respectively, Fig. 4B).

**Table 2:** Univariate and multivariate analyses of factors associated with survival and recurrence in 327 HCCs.

Variables	OS				Cumulative recurrence			
	Univariate, *P*	Multivariate			Univariate, *P*	Multivariate		
		HR	95%CI	*p* value		HR	95%CI	*p* value
Sex (male vs. female)	0.767			NA	0.399			NA
Age, years (<52 vs. ≥52)	0.253			NA	0.691			NA
HBsAg (negative vs. positive)	0.535			NA	0.208			NA
Liver cirrhosis (yes vs. no)	0.615			NA	0.387			NA
Preoperative treatment (yes vs. no)	0.140			NA	0.203			NA
Child–Pugh score (A vs. B)	0.169			NA	0.152			NA
Serum AFP (≤20 vs. >20 ng/mL)	0.028			NS	0.323			NA
Tumor diameter (>5 vs. ≤5 cm)	<0.001	1.377	1.021–1.856	0.036	0.015	1.414	1.038–1.926	0.028
Tumor number (multiple vs. single)	0.001	1.782	1.244–2.552	0.002	0.009	1.707	1.146–2.541	0.008
Microvascular invasion (yes vs. none)	<0.001	1.878	1.162–3.035	0.010	0.002	2.196	1.322–3.650	0.002
Tumor encapsulation (none vs. complete)	<0.001			NS	0.083			NA
Tumor differentiation (I/II vs. III/IV))	0.018			NS	0.070			NA
TNM stage (I/II vs. III)	<0.001			NA	<0.001			NA
LRP1 expression (low vs. high)	<0.001	1.484	1.100–2.003	0.010	0.015	1.406	1.032–1.916	0.031

Abbreviations and Note: OS, overall survival; NA, not adopted; NS, not significant; AFP, α-fetoprotein; HBsAg, hepatitis B surface antigen; TNM, tumor-node-metastasis; 95%CI, 95% confidence interval; HR, Hazard ratio; Cox proportional hazards regression model.

### Correlation between LRP1 and MMP9 Expression in Patient Outcome

Previously, we identified that MMP9 expression was positively correlated with poor prognosis of HCC patients in the same cohort [21]. The MMP9 protein was observed in cytoplasm of cells, stromal fibroblasts and inflammatory cells (Fig. 3I, J, K and L). Spearman's correlation analysis showed a negative correlation between LRP1 and MMP9 expression (*r* = −0.291, P<0.001). Furthermore, we also investigated the effect of combined LRP1 and MMP9 expression on patient outcome. Patients were divided into four subgroups: (I) LRP1^high^/MMP9^high^ (n = 56), (II) LRP1^low^/MMP9^high^ (n = 108), (III) LRP1^high^/MMP9^low^ (n = 103), and (IV) LRP1^low^/MMP9^low^ (n = 60). The HCC patients with LRP1^low^/MMP9^high^ had the worst prognosis among the four subgroups (Fig. 4C and D).

### Independent validation

Low level of LRP1 predicted an unfavorable prognosis in the validation set containing 161 HCC patients (Fig. S1). The prognostic value of LRP1 expression was validated in independent data set using Cox proportional hazards model analysis, and the results of which demonstrated that LRP1 was an independent prognostic indicator for OS (P = 0.001) and cumulative recurrence (P = 0.010, Table S2).

## Discussion

The present study described that LRP1 was lowly expressed in HCC cell lines as well as in HCC specimens, consistent with the expression of low-density lipoprotein receptor previously reported in HCC cells [26]. Although we failed to construct a plasmid that overexpressed LRP1, owing to its large molecular mass (∼600 kDa) [5], our results still provided powerful evidence to support that high expression of LRP1 was associated with low metastatic ability of HCC both in vivo and vitro. More importantly, we addressed low level of LRP1 had unfavorably prognostic implication in the 2 independent cohorts of HCC patients.

Although several studies have implicated LRP1 in tumorigenesis, its precise role and potential underlying mechanisms remain controversial. For example, several reports have shown that low expression of LRP1 is closely related to the aggressive phenotype of tumor cells derived from various tissues, such as human prostate, thyroid, and breast cancer [9], [27]. However, other studies identified that inhibition of LRP1 expression and function decreased cell migration and invasion [14], [28], [29]. Therefore, we consider that the LRP1 function in tumor cell migration and invasion may depend on the tumor cell type and the specific extracellular proteins involved in these processes. In our institute, quantitative proteomics analysis of metastasis-related proteins in HCC has shown an enhanced expression of LRP1 in MHCC97L cells (with low metastasis potential) compared with MHCC97H cells (with high metastasis potential) [15]. Here, we further showed that the low level of LRP1 in HCC cells associated with the metastatic potential of HCC cells. First, we found that HCC cells expressing low LRP1 were tend to have high metastatic potential. Second, after down-regulation of LRP1 expression in low-metastatic SMMC-7721 cells, the cells showed significantly increased migration and invasion in vivo and in vitro. In particular, clinical data demonstrated that malignant pathological phenotypes were more frequent in patients with low LRP1 expression than those with high expression. Moreover, LRP1 expression was independent of other prognostic markers (large tumor size, microvascular invasion, and multiple tumors) for both OS and cumulative recurrence. So we draw the conclusion that low expression of LRP1 does promote the metastasis and invasion of HCC and may be a prognostic indicator for HCC.

LRP1 was first characterized as an endocytic receptor for apolipoprotein-E-containing lipoprotein particles and for α2-macroglobulin. Since then, >40 ligands have been identified, including proteases, protease inhibitors, growth factors, extracellular matrix proteins, and foreign toxins. By binding bifunctional extracellular ligands and intracellular signaling-adaptor proteins, LRP1 may promote the internalization and catabolism of other receptors with cell signaling activity [30]. By binding adaptor proteins, LRP1 also directly regulates the activity of various cell signaling enzymes, including ERK/MAP kinase, PI3K, and c-Jun NH2-terminal protein kinase [5]. The diverse activities of LRP1 suggest a model in which this receptor functions as a “sensor” of the cellular microenvironment. Such activity should be highly relevant to cancer because it is now widely accepted that a tumor and its microenvironment actively and reciprocally interact at all stages of cancer progression. To determine how LRP1 promotes HCC cell migration and invasion, we focused on elucidating the relationship between LRP1 and MMPs which has been reported to participate closely in tumor progression in HCC [31]. Here, we found that LRP1 silencing significantly increased the expression and bioactivities of MMP9 in SMMC-7721 cells. Our results also showed a negative correlation between LRP1 and MMP9 protein expression by correlation analysis in HCC tissues, though we did not detect MMP9 activity owing to the lack of effective measures. The extracellular subunit (α-chain) of LRP1 harbors four ligand-binding clusters that are involved in the specific recognition of extracellular ligands [32]. Recently, multiple lines of evidence have shown a tight link between LRP1 and MMPs [33]. Furthermore, recent study reported MMPs may be modulated by their cellular receptors that mediate their rapid internalization and degradation [5]. Direct evidence provided by Hahn–Dantona et al. has revealed that cell lines genetically deficient in LRP1 have diminished capacity to mediate catabolism of MMP9, and the assays in vitro have demonstrated the direct high-affinity interaction between MMP9 and LRP1 [34]. Our previous study showed the importance of MMP9 regulation in HCC, moreover, it involved a variety of processes associated with progression and metastasis of HCC [21]. Thus, we propose that LRP1 might regulate tumor migration and invasion by altering the level of MMP9.

In general, LRP1 modulates MMP9 expression and low level of LRP1 in HCC cells is associated with tumor aggressiveness in HCC. Low level of LRP1 predicts an unfavorable prognosis of HCC after curative resection. LRP1 may offer a possible strategy for tumor molecular therapy.

## Supporting Information

Figure S1 **Prognostic implication was assessed by Kaplan–Meier analysis and log-rank tests in validation set consisting of 161 HCC patients.** HCC patients with high LRP1 expression had better prognosis in terms of overall survival (**A**) and cumulative recurrence (**B**).
(TIF)

Table S1 **Clinicopathologic features of the 161 HCCs.**
(DOC)

Table S2 **Univariate and multivariate analyses of factors associated with survival and recurrence in 161 HCCs.**
(DOC)
