# Supplementary figures and images for: Low Level of Low-Density Lipoprotein Receptor-Related Protein 1 Predicts an Unfavorable Prognosis of Hepatocellular Carcinoma after Curative Resection
Source: PLoS One. 2012 Mar 12;7(3):e32775. doi: 10.1371/journal.pone.0032775 (PMC3299691; doi:10.1371/journal.pone.0032775)

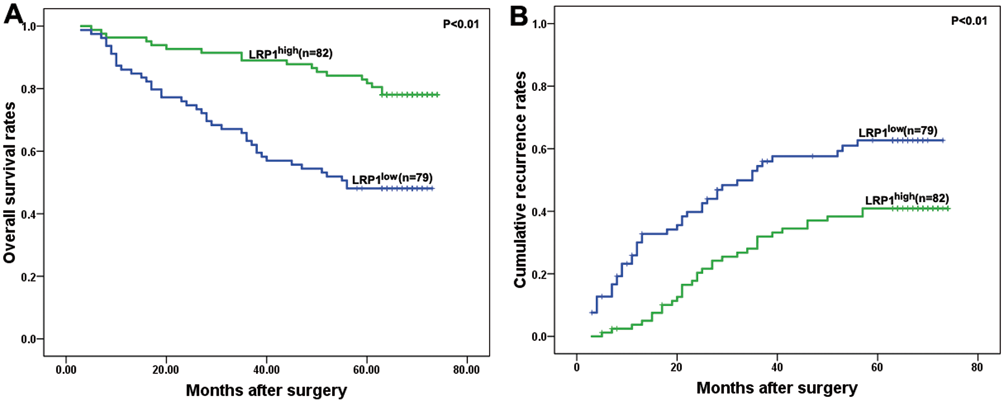

Supplement: Figure S1 — Prognostic implication was assessed by Kaplan–Meier analysis and log-rank tests in validation set consisting of 161 HCC patients. HCC patients with high LRP1 expression had better prognosis in terms of overall survival (A) and cumulative recurrence (B). (TIF) [file pone.0032775.s001.tif]
