# Supplementary material for: Low Level of Low-Density Lipoprotein Receptor-Related Protein 1 Predicts an Unfavorable Prognosis of Hepatocellular Carcinoma after Curative Resection
Source: PLoS One. 2012 Mar 12;7(3):e32775. doi: 10.1371/journal.pone.0032775 (PMC3299691; doi:10.1371/journal.pone.0032775)
Supplement: Table S1 — Clinicopathologic features of the 161 HCCs. (DOC) [file pone.0032775.s002.doc]

**Table S1 Clinicopathologic features of the 161 HCCs**

| variables | Results |
| --- | --- |
| Gender (male/female)  Age (≤50y/>50y)  HBsAg (Positive/Negative)  Anti-HCVAb (Positive/Negative)  AFP (≤20/>20, ng/ml)  Serum ALT (≤75/>75, U/L)  Liver cirrhosis (yes/no)  Tumor diameter (≤5/>5,cm)  Tumor number (single/multiple)  Microvascular invasion (yes / none)  Tumor encapsulation (complete/none)  Tumor differentiation (I-II / III-IV)  TNM (I-II/III) | 140/21  88/73  140/21  8/153  52/109  142/19  123/38  93/68  141/20  44/117  99/62  122/39  105/56 |

Abbreviations: AFP, α-fetoprotein; HBsAg, hepatitis B surface antigen; HCV, hepatitis C virus;

ALT, Alanine aminotransferase; TNM, tumor-node-metastasis
