# Supplementary material for: Low Level of Low-Density Lipoprotein Receptor-Related Protein 1 Predicts an Unfavorable Prognosis of Hepatocellular Carcinoma after Curative Resection
Source: PLoS One. 2012 Mar 12;7(3):e32775. doi: 10.1371/journal.pone.0032775 (PMC3299691; doi:10.1371/journal.pone.0032775)
Supplement: Table S2 — Univariate and multivariate analyses of factors associated with survival and recurrence in 161 HCCs. (DOC) [file pone.0032775.s003.doc]

**Table S2 Univariate and multivariate analyses of factors associated with survival and recurrence in 161 HCCs**

| Variables | OS | | | | Cumulative recurrence | | | |
| --- | --- | --- | --- | --- | --- | --- | --- | --- |
|  | Univariate, *P* | Multivariate | | | Univariate, *P* | Multivariate | | |
|  |  | HR | 95%CI | *P* value |  | HR | 95%CI | *P* value |
| Sex (male *vs.* female) | 0.657 |  |  | NA | 0.087 |  |  | NA |
| Age, years (≤50y *vs.* >50y) | 0.104 |  |  | NA | 0.116 |  |  | NA |
| HBsAg (positive *vs.* negative) | 0.288 |  |  | NA | 0.843 |  |  | NA |
| HCVAb(positive *vs.* negative) | 0.448 |  |  | NA | 0.686 |  |  | NA |
| Serum ALT(≤75 U/L *vs.* >75 U/L) | 0.481 |  |  | NA | 0.754 |  |  | NA |
| Liver cirrhosis (yes *vs.* no) | 0.676 |  |  | NA | 0.836 |  |  | NA |
| Serum AFP (≤20 ng/mL *vs.* > 20 ng/mL) | 0.334 |  |  | NA | 0.797 |  |  | NA |
| Tumor diameter (≤5cm *vs.* >5cm) | 0.003 | 0.454 | 0.267-0.771 | 0.003 | 0.001 | 0.482 | 0.303-0.765 | 0.002 |
| Tumor number (multiple *vs.* single) | <0.001 | 5.060 | 2.804-9.132 | <0.001 | 0.015 | 2.119 | 1.061-4.230 | 0.033 |
| Microvascular invasion (yes *vs.* none) | <0.001 | 1.844 | 1.073-3.170 | 0.027 | <0.001 | 1.919 | 1.168-3.155 | 0.010 |
| Tumor encapsulation (none *vs.* complete ) | 0.740 |  |  | NA | 0.779 |  |  | NA |
| Tumor differentiation (I/II *vs.* III/IV) ) | 0.266 |  |  | NA | 0.334 |  |  | NA |
| TNM stage (I/II *vs.* III) | 0.001 |  |  | NA | 0.001 |  |  | NA |
| LRP1 expression (high *vs.* low) | <0.001 | 0.381 | 0.231-0.681 | 0.001 | 0.002 | 0.532 | 0.329-0.859 | 0.010 |

Abbreviations and Note: OS, overall survival; NA, not adopted; NS, not significant; AFP, α-fetoprotein; HBsAg, hepatitis B surface antigen; HCV, hepatitis C virus; 95%CI, 95% confidence interval; HR, Hazard ratio; Cox proportional hazards regression model.
